# Family history of psychiatric disorders as a risk factor for maternal postpartum depression: a systematic review protocol

**DOI:** 10.1186/s13643-022-01952-1

**Published:** 2022-04-15

**Authors:** Mette-Marie Zacher Kjeldsen, Alessio Bricca, Xiaoqin Liu, Vibe G. Frokjaer, Kathrine Bang Madsen, Trine Munk-Olsen

**Affiliations:** 1 National Centre for Register-based Research, Aarhus University, Fuglesangs Allé 26, 8210 Aarhus V, Aarhus, Denmark; 2 Department of Public Health, Aarhus University, Aarhus, Denmark; 3 Research Unit for Musculoskeletal Function and Physiotherapy, Department of Sports Science and Clinical Biomechanics, University of Southern Denmark, Odense, Denmark; 4 The Research Unit PROgrez, Department of Physiotherapy and Occupational Therapy, Næstved-Slagelse-Ringsted Hospitals, Slagelse, Denmark; 5 Neurobiology Research Unit, Copenhagen University Hospital – Rigshospitalet, Copenhagen, Denmark; 6 Mental Health Services, Capital Region of Denmark, Copenhagen, Denmark; 7 Faculty of Health and Medical Sciences, University of Copenhagen, Copenhagen, Denmark; 8 Department of Clinical Research, University of Southern Denmark, Odense, Denmark

**Keywords:** Postpartum depression, Postnatal depression, Family psychiatry, Psychiatric family history, Familial risk, Risk factor, Meta-analysis, PRISMA-P

## Abstract

**Background:**

Postpartum depression (PPD) is the most common postpartum psychiatric disorder, affecting 11–15% of new mothers, and initiatives towards early identification and treatment are essential due to detrimental consequences. Family history of psychiatric disorders is a risk factor for developing psychiatric episodes outside the postpartum period, but evidence of the association between familial risk and PPD is not clear. Hence, the objective of this systematic review is to summarize the current literature on the association between family history of psychiatric disorders and PPD.

**Methods:**

This protocol has been developed and reported according to the PRISMA-P guidelines for systematic reviews. A comprehensive literature search will be conducted in PubMed, Embase, and PsycINFO from inception of the databases, supplemented with citation tracking and reference screening of the included studies. Two independent authors will examine all retrieved articles for inclusion in two steps: title/abstract screening and full-text screening. Eligible studies are case-control and cohort studies reporting a risk estimate for the association between family history of psychiatric disorders and PPD. Studies will be assessed for risk of bias using the Newcastle-Ottawa Scale. The association between family psychiatry and PPD will be combined in a meta-analysis using a restricted maximum likelihood method (REML). Heterogeneity will be quantified using I^2^ and investigated through meta-regression, subgroup and sensitivity analyses, and publication bias will be evaluated via visual inspection of a funnel plot. The overall strength and quality of the findings will be evaluated using the GRADE (Grading of Recommendations Assessment, Development, and Evaluation) approach. If meta-analysis is not possible, data will be synthesized narratively in text and tables.

**Discussion:**

This systematic review will be the first to summarize current knowledge and present an overall estimate for the association between family history of psychiatric disorders and PPD. Evaluation of psychiatric family history as a PPD risk factor is essential to assist early identification of women at high risk of PPD in routine perinatal care.

**Systematic review registration:**

PROSPERO ID: 277998 (registered 10th of September 2021).

## Background

The postpartum period is considered a major life transition associated with emotional, physical, and social changes [1]. There is substantial evidence that childbirth increases the risk of mental disorders in women, including diagnoses of unipolar depressive disorders, bipolar affective disorders, adjustment disorders, schizophrenia, and psychosis [2]. The most common postpartum psychiatric disorder is postpartum depression (PPD), with a prevalence of 11-15%, depending on the population under investigation and definition [1, 3–5]. Definition of PPD in research settings ranges from a strict clinical diagnosis according to DSM-V (applying a specifier indicating onset within 4 weeks postpartum) or ICD-10 (onset within 6 weeks postpartum) to less strict definitions with the use of screening tools such as The Edinburgh Postnatal Depression Scale (EPDS) or measures of psychiatric treatment with the onset period varying from a few weeks and up to 12 months postpartum [4, 5]. Most new mothers recover from PPD within a few months, but approximately 25-30% still suffer from PPD one year after the delivery [6]. Negative consequences of PPD include maternal mortality, child behavioral, health-related (e.g., asthma and higher rates of injuries) and cognitive development problems, and compromised mother-baby interaction and attachment [5, 7, 8]; all of which have a detrimental impact on the mother, the child, and the entire family, as well as considerable societal costs.

Identification of PPD is possible in primary care and community settings with the use of screening tools where the EPDS is the most widespread screening tool used to identify women at risk of PPD [9, 10]. The tool is widely used to identify women who could be referred for further diagnostic evaluation and administered by healthcare professionals in community settings, e.g., at health nurse home visits in the postpartum period. Treatment of PPD has shown to be feasible and effective, in particular counseling-based interventions, such as cognitive behavioral therapy and interpersonal therapy, but also antidepressant medication [11–14].

Family history of psychiatric disorders is a well-known risk factor for developing psychiatric disorders outside the postpartum period [15–18]. Within the postpartum period, familial risk has similarly been identified as a risk factor for postpartum psychiatric disorders in family studies; having a close relative with a previous postpartum mood disorder more than doubles the risk of developing a postpartum mood disorder [19–22]. Adding to this, results indicate PPD is more heritable than major depressive disorder outside the postpartum period [23]. A few other studies investigating a range of different risk factors have also identified family history of psychiatric disorders as a risk factor for PPD [24–26]. However, several systematic reviews and umbrella reviews of risk factors for PPD do not identify family psychiatry as a significant risk factor for PPD [27–34].

Despite solid knowledge that family history of psychiatric disorders is a strong risk factor for developing psychiatric disorders outside the postpartum period, to the best of our knowledge, no systematic review has summarized current knowledge on family psychiatry as a risk factor for PPD in women.

### Objective

This systematic review summarizes the current literature on the relationship between family history of psychiatric disorders and PPD within 12 months postpartum. Secondary, the aim is to investigate the association between familial risk and PPD stratified on different definitions of the exposure and the outcome, respectively.

## Methods

This protocol is conducted following the PRISMA-P guidelines for systematic review protocols [35, 36]. The protocol has been registered with the International Prospective Register of Systematic Reviews (PROSPERO) on the 10th of September 2021 (ID nr. 277998). In case of future amendments to this protocol, a description of the change and rationale will be specified in the final publication, and the PROSPERO protocol will be updated with the amendments.

### Eligibility criteria

Studies must meet the following eligibility criteria:

#### Participants

Studies of mothers at any age who have given birth with no restriction to parity, will be included. A postpartum period of up to 12 months will be considered, and if a study has followed participants longer than 12 months from giving birth, the study will only be included if the authors have either reported a relative risk estimate, or sufficient data to calculate one, separately within the first 12 months postpartum.

#### Exposure

Eligible studies include prospective and retrospective studies investigating family history of psychiatric disorders as a risk factor for PPD. Including studies investigating several risk factors for PPD, where family psychiatry is one of them. Family should be defined as any family member (parents, siblings, or other biological family members). Psychiatric history should be any psychiatric illness (excluding alcohol and drug abuse) at any time prior to the birth of the index child. Information on family psychiatry could be obtained through registers, medical records, or self-reported. A self-reported “yes/no” question to family psychiatry would be sufficient for a study to be included.

#### Outcome

Studies must have studied PPD with an onset within 12 months from birth as one of the outcomes. PPD could be identified through registers (using codes in accordance or similar to F32, F33, and F34.1 in ICD-10 codes or prescription of antidepressant medication), medical records, clinical interviews, or using validated instruments (e.g., EPDS or Beck Depression Inventory (BDI)). If a study assessed the perinatal period or both the antenatal and postnatal period, inclusion is conditional on a clear distinction in the reporting, so that they have reported separately for the postpartum period.

#### Study design

Only peer-reviewed published cohort and case-control studies will be included. All other study designs will be excluded (e.g., case reports, RCTs, systematic reviews, cross-sectional studies, editorials, letters, thesis).

#### Setting and geography

There will be no restriction to the setting and no restriction on geography.

#### Language and publication year

Included studies will be restricted to studies reported in English, Danish, Norwegian, Swedish, and Italian, and there will be no restriction on publication year.

### Information sources and search strategy

An extensive search will be performed in the electronic databases PubMed, Embase, and PsycINFO from inception of the databases. This search will be supplemented with citation tracking in Web of Science and reference screening of the included studies.

The search strings have been constructed combining text words and thematic, index words (MeSH terms in PubMed, Emtree words in Embase, and thesaurus words in PsycINFO). The search words have been organized in blocks focusing on “Family history of psychiatric disorders” and “PPD”. The family psychiatry block consists of three themes: *family*, *psychiatry*, and *history*. Within each theme, e.g., all words related to family, the text words and thematic index words have been combined with the Boolean operator *OR* and all three themes have then been combined with the Boolean operator *AND*. Thematic index words covering the entire theme, e.g., the MeSH term *family health*, have then been combined in the search string with an *OR*. The same has been done for the PPD block containing two themes: *postpartum* and *depression*. The two blocks search strings are combined with an *AND*. All three search strings are displayed in Table 1.

**Table 1:** Search strings

Database	Search string
PubMed	(((famil*[Title/Abstract] OR paren*[Title/Abstract] OR proband*[Title/Abstract] OR relative [Title/Abstract] OR mother[Title/Abstract] OR father[Title/Abstract] OR sister[Title/Abstract] OR brother[Title/Abstract] OR family[MeSH Terms] OR parents[MeSH Terms] OR siblings[MeSH Terms] OR mothers[MeSH Terms] OR fathers[MeSH Terms]) AND (psychiat*[Title/Abstract] OR mental disorder*[Title/Abstract] OR psychopath*[Title/Abstract] OR psychiatry[MeSH Terms] OR mental disorders[MeSH Terms] OR psychopathology[MeSH Terms]) AND (histor*[Title/Abstract] OR history[MeSH Terms])) OR (medical history taking[MeSH Terms] OR family health[MeSH Terms])) AND (((postpartum[Title/Abstract] OR postnat*[Title/Abstract] OR post-nat*[Title/Abstract] OR perinat*[Title/Abstract] OR postpartum period[MeSH Terms]) AND (depress*[Title/Abstract] OR episod*[Title/Abstract] OR disorder[Title/Abstract] OR psychiat*[Title/Abstract] OR psychopat*[Title/Abstract] OR depression[MeSH Terms] OR depressive disorder[MeSH Terms] OR psychiatry[MeSH Terms] OR psychopathology[MeSH Terms])) OR (depression, postpartum[MeSH Terms]))
Embase	(((famil*:ab,ti OR paren*:ab,ti OR proband*:ab,ti OR relative:ab,ti OR mother:ab,ti OR father:ab,ti OR sister:ab,ti OR brother:ab,ti OR ‘family’/exp OR ‘parent’/exp OR ‘sibling’/exp OR ‘mother’/exp OR ‘father’/exp) AND (psychiat*:ab,ti OR mental disorder*:ab,ti OR psychopath*:ab,ti OR ‘psychiatry’/exp OR ‘mental disease’/exp) AND (histor*:ab,ti OR ‘history’/exp)) OR (‘family health’/exp OR ‘family history’/exp)) AND (((postpartum:ab,ti OR postnat*:ab,ti OR post-nat*:ab,ti OR perinat*:ab,ti OR ‘puerperium’/exp) AND (depress*:ab,ti OR episod*:ab,ti OR disorder:ab,ti OR psychiat*:ab,ti OR psychopat*:ab,ti OR ‘depression’/exp OR ‘psychiatry’/exp)) OR (‘postnatal depression’/exp))
PsycINFO	((((ab(famil*) OR ab(paren*) OR ab(proband*) OR ab(relative) OR ab(mother) OR ab(father) OR ab(sister) OR ab(brother) OR MAINSUBJECT.EXACT(“Family”) OR MAINSUBJECT.EXACT(“Parents”) OR MAINSUBJECT.EXACT(“Siblings”) OR MAINSUBJECT.EXACT(“Mothers”) OR MAINSUBJECT.EXACT(“Fathers”)) AND (ab(psychiat*) OR ab(mental disorder*) OR ab(psychopath*) OR MAINSUBJECT.EXACT(“Psychiatry”) OR MAINSUBJECT.EXACT(“Mental Disorders”) OR MAINSUBJECT.EXACT(“Psychopathology”)) AND (ab(histor*) OR MAINSUBJECT.EXACT(“History”))) OR (MAINSUBJECT.EXACT(“Family History”))) AND (((ab(postpartum) OR ab(postnat*) OR ab(post-nat*) OR ab(perinat*) OR MAINSUBJECT.EXACT(“Perinatal Period”)) AND (ab(depress*) OR ab(episod*) OR ab(disorder) OR ab(psychiat*) OR ab(psychopat*) OR MAINSUBJECT.EXACT(“Depression (Emotion)”) OR MAINSUBJECT.EXACT(“Psychiatry”) OR MAINSUBJECT.EXACT(“Psychopathology”))) OR MAINSUBJECT.EXACT(“Postpartum Depression”)))

The search strings have been reviewed by a professional health science librarian for input to optimize the searches throughout the process of developing the search strings.

Before finalizing the review manuscript, the literature search will be re-run in all three databases to identify potential newly published papers not yet retrieved and screened for inclusion.

### Selection process and study records

Duplicates will be removed in EndNote, and afterward, the search results will be uploaded to Covidence, an online tool to be used for screening titles and abstracts [37, 38]. Two authors (MZK and KBM) will independently screen titles and abstracts for eligibility. All studies judged as meeting the eligibility criteria by at least one author will be full text screened for inclusion. In case of disagreement, a third member of the review team will be drawn into the discussion. Throughout full-text screening, reasons for exclusion of papers will be recorded in an Excel spreadsheet. The number of studies retrieved, reviewed, included, and excluded will be reported in a flowchart in accordance with the PRISMA recommendation.

To “calibrate” the two review authors before the selection process, a minor training session will be held. Both authors will evaluate the first 50 articles (first title/abstract, then full text when title/abstract indicates relevance of the study) and compare their included and excluded studies on each of the two selection stages. If inconsistencies occur, the reviewers will discuss this to ensure similar selection of studies.

### Extraction of data and data items

Data extraction will be done independently by two authors (MZK and KBM) using predefined data extraction forms in Excel developed for this systematic review. Any disagreement will be resolved by discussion. Before extracting the data, both authors will do a pilot of the predefined forms for data extraction on five studies. After this, they will compare their extractions and discuss any disagreement.

The following variables will be extracted:

- Study identification and description: authors, publication year, study title, journal, source country/continent/site, recruitment and follow-up period, and study objective.
- Population and participants: total sample size, participation/response rate, distribution in groups (distribution of cases/non-cases in relation to exposure/non-exposure), sample characteristics (e.g., mean age, education/income/socioeconomic status, ethnicity), inclusion/exclusion criteria, and PPD prevalence/incidence.
- Exposure: definition of family and how information on familial psychiatric status was obtained. Information on family member with (current or previous) psychiatric disorder.
- Outcome: definition of PPD and how information on PPD was obtained, other relevant information (e.g., which register/validated instrument, cut-off on validated instrument, time of assessment, mean value)
- Study design: (prospective or retrospective) cohort study or case-control study.
- Statistical analysis and findings: statistical approach, adjustments for confounding, risk estimate for the association between family history of psychiatric disorders and PPD.

The primary outcome of this systematic review is to investigate the association between family history of psychiatric disorders and PPD. Therefore, risk estimates on the association in the included studies will be presented and an overall estimate will be calculated through meta-analysis.

The secondary aim is to investigate the impact of different definitions of the exposure and outcome on the cumulative estimate. First, the association between familial risk and PPD will be stratified on different time points for assessment of PPD (e.g., 3, 6, 9, and 12 months postpartum) and on different ways of defining PPD (registers, medical records, clinical interviews, or validated instruments). Secondly, the association will be stratified on the different definitions of family psychiatry (information obtained through registers, medical records, or self-reported).

If the association is presented for several time points in the included study, estimates for all time points will be extracted and the time point closest to birth will be used in the main analysis. The other estimates will be included in the secondary analysis investigating the definition of the outcome.

In case the same study population is included in more than one study, all studies will be included in the descriptive tables, but the overlap will be marked in the reporting of results, and the population will only be included once in the meta-analysis to avoid double counting [39].

In case of missing data, the corresponding author of the study will be contacted through email requesting for the necessary data. If we experience problems with delivering the email, the second author will be contacted and so forth. After three days without a response from the author, we will send a reminder to the corresponding author and the last author. After further seven days without a response, we will resend the email to the corresponding and last author. If the authors do not reply or if the relevant information is not available, the studies will not be included in the meta-analysis.

### Assessment of risk of bias

Assessment of risk-of-bias in the included studies will be done using the Newcastle-Ottawa Scale specifically for cohort and case-control studies [40]. The scale consists of eight items covering three dimensions: selection, comparability, and either outcome for cohort studies or exposure for case-control studies. Each item can be given a maximum of one star if the quality is high, and a maximum of two stars can be given to comparability, totaling a maximum of 9 stars for the studies of the highest quality.

The risk assessment will be performed independently by two authors (MZK and KBM). In situations with disagreement, consensus will be reached via discussion. To ensure the two authors make similar risk assessments, they will assess the first five included studies and discuss discrepancies in the assessment before assessing the rest of the included studies. The risk-of-bias assessment score will be included in the outcome table to visualize the strength of each study and further investigated in meta-regression and sensitivity analyses.

### Data synthesis

Characteristics of the included studies will be presented in a table. The characterization will be based on a description of the population, exposure, outcome, study design, and findings supplemented with the risk of bias assessment score.

Meta-analysis will be conducted to summarize the association between family history of psychiatric disorders and PPD with an odds ratio (OR). If the association between family psychiatry and PPD is presented as an OR, this will be extracted. If both unadjusted and adjusted estimates are presented, the adjusted estimates will be used. If a risk estimate for the association is not presented, but sufficient data to estimate an OR is (distribution of participants in relation to case status (having PPD or not) and exposure status (having family psychiatric history or not)) these data will be extracted to estimate the OR. If a risk ratio (RR) is presented together with the prevalence of PPD among non-exposed participants, this will be extracted, and the RR will be adjusted to an OR according to guidelines in the Cochrane Handbook [41]. If sufficient data to estimate an OR or adjust another risk estimate to an OR is not presented, the authors will be contacted to obtain this information. Meta-analysis will then be done with ORs in a random-effects model with the use of restricted maximum likelihood (REML) method. This will be illustrated in a forest plot.

Statistical heterogeneity will be evaluated using *Q*-test and quantified with the *I*^2^ ranging from 0% (no heterogeneity) to 100% (most heterogeneity) [42]. Heterogeneity will be investigated through meta-regression, subgroup and sensitivity analyses (e.g., investigating the impact of risk-of-bias assessment scores, study design, definition of exposure and outcome, unadjusted/adjusted estimates, and geography). Publication bias will be investigated through a visual inspection of the funnel plot looking for asymmetry as an indication of potential small study bias [43]. As we only include case-control and cohort studies, we do not expect to be able to identify prespecified protocols on the included primary studies, and therefore, we will not investigate selective reporting bias through published protocols [44]. Stata version 17 will be used to perform the meta-analysis.

Meta-analysis will be done, if at least two studies are included with sufficient information to make meta-analysis. If meta-analysis is not feasible, the findings will be summarized in text and tables focusing on the association between family history of psychiatric disorders and PPD in the included studies using the synthesis without meta-analysis guidelines [45].

### Overall quality of evidence

The overall quality of the evidence will be assessed using the GRADE (Grading of Recommendations Assessment, Development, and Evaluation) recommendation for prognostic studies as we will include observational studies with a broadly defined population [46, 47]. The quality of the evidence from observational studies starts as high quality and can be either downgraded or upgraded. Downgrading is based on five domains focusing on methodological flaws in the included studies; study limitations, inconsistency, indirectness, imprecision, or indications of publication bias, whereas upgrading is based on two domains of methodological strengths; a large effect or a dose-response relationship. Based on this, confidence in the estimate will be judged as either; high, moderate, low, or very low [46, 47].

## Discussion

The discussion will focus on strengths and limitations of the current systematic review.

As there is no fixed definition or ways of measuring PPD, it is seen as a major strength that all definitions of PPD, also relatively broad definitions (registers, medical records, clinical interviews, and validated screening instruments) will be included, and will be further investigated in subgroup analysis in the meta-analysis. Additionally, the search strings to be used for the comprehensive search in three databases from inception until the date of the final database search were developed with input from a health librarian, which also serves as a strength of this review. Two authors will independently perform the study selection, data extraction, and risk of bias assessment of the included studies, and before each of these steps, the authors will make small training sessions to make selection, extraction, and assessment similar, which is also seen as a methodological strength. Lastly, the transparent and systematic methods used when following the PRISMA-P guidelines and the pre-specification of methods in a protocol are also seen as a clear advantage of this systematic review.

Only English, Danish, Swedish, Norwegian, and Italian peer-reviewed, published papers will be included, which may lead to exclusion of relevant papers in other languages or not published papers. This might potentially limit the generalizability of the findings and validity of the estimate, but potential publication bias will be investigated using funnel plot looking for asymmetry to identify small study bias. Also, studies excluded due to language will be reported in the flowchart showing the magnitude of the excluded literature.

## Data Availability

Please contact first author, Mette-Marie Zacher Kjeldsen, for enquiries related to the dataset and STATA script.

## Abbreviations

BDI: Beck Depression Inventory
EPDS: Edinburgh Postnatal Depression Scale
GRADE: Grading of Recommendations Assessment, Development, and Evaluation
OR: Odds ratio
PPD: Postpartum depression
PROSPERO: International Prospective Register of Systematic Reviews
REML: Restricted maximum likelihood method
RR: Risk ratio/relative risk

## References

[1] Howard LM, Molyneaux E, Dennis CL, Rochat T, Stein A, Milgrom J (2014). Non-psychotic mental disorders in the perinatal period. Lancet..

[2] Munk-Olsen T, Laursen TM, Pedersen CB, Mors O, Mortensen PB (2006). New Parents and Mental Disorders. Jama..

[3] Gavin N, Gaynes B, Lohr K, Meltzer-Brody S, Gartlehner G, Swinson T (2005). Perinatal depression: a systematic review of prevalence and incidence. Obstet Gynecol.

[4] Wisner KL, Moses-Kolko EL, Sit DKY (2010). Postpartum depression: a disorder in search of a definition. Arch Womens Ment Health.

[5] O’Hara MW, Mc Cabe JE (2013). Postpartum depression: current status and future directions. Annu Rev Clin Psychol.

[6] Goodman J (2004). Postpartum depression beyond the early postpartum period. J Obstet Gynecol Neonatal Nurs.

[7] Brummelte S, Galea LAM (2016). Postpartum depression: etiology, treatment and consequences for maternal care. Horm Behav.

[8] Pierce M, Hope HF, Kolade A, Gellatly J, Osam CS, Perchard R (2020). Effects of parental mental illness on children’s physical health: systematic review and meta-analysis. Br J Psychiatry.

[9] Meltzer-Brody S, Howard LM, Bergink V, Vigod S, Jones I, Munk-Olsen T (2018). Postpartum psychiatric disorders. Nat Rev Dis Primers.

[10] Cox JL, Holden JM, Sagovsky R (1987). Detection of postnatal depression. Development of the 10-item Edinburgh Postnatal Depression Scale. Br J Psychiatry.

[11] Vigod SN, Wilson CA, Howard LM (2016). Depression in pregnancy. BMJ..

[12] Brown JVE, Wilson CA, Ayre K, Robertson L, South E, Molyneaux E (2021). Antidepressant treatment for postnatal depression. Cochrane Database Syst Rev.

[13] Sockol LE (2015). A systematic review of the efficacy of cognitive behavioral therapy for treating and preventing perinatal depression. J Affect Disord.

[14] O’Connor E, Senger CA, Henninger ML, Coppola E, Gaynes BN (2019). Interventions to prevent perinatal depression: evidence report and systematic review for the US Preventive Services Task Force. JAMA..

[15] Laursen TM, Labouriau R, Licht RW, Bertelsen A, Munk-Olsen T, Mortensen PB (2005). Family history of psychiatric illness as a risk factor for schizoaffective disorder: a Danish register-based cohort study. Arch Gen Psychiatry.

[16] Lichtenstein P, Yip BH, Björk C, Pawitan Y, Cannon TD, Sullivan PF (2009). Common genetic influences for schizophrenia and bipolar disorder: a population-based study of 2 million nuclear families. Lancet..

[17] Wray NR, Gottesman II (2012). Using summary data from the Danish National Registers to estimate heritabilities for schizophrenia, bipolar disorder, and major depressive disorder. Front Genet.

[18] Sullivan PF, Neale MC, Kendler KS (2000). Genetic epidemiology of major depression: review and meta-analysis. Am J Psychiatry.

[19] Murphy-Eberenz K, Zandi PP, March D, Crowe RR, Scheftner WA, Alexander M (2006). Is perinatal depression familial?. J Affect Disord.

[20] Payne JL, Mackinnon DF, Mondimore FM, Mcinnis MG, Schweizer B, Zamoiski RB (2008). Familial aggregation of postpartum mood symptoms in bipolar disorder pedigrees. Bipolar Disord.

[21] Kimmel M, Hess E, Roy PS, Palmer JT, Meltzer-Brody S, Meuchel JM (2015). Family history, not lack of medication use, is associated with the development of postpartum depression in a high-risk sample. Arch Womens Ment Health.

[22] Forty L, Jones L, Macgregor S, Caesar S, Cooper C, Hough A (2006). Familiality of postpartum depression in unipolar disorder: results of a family study. Am J Psychiatry.

[23] Viktorin A, Meltzer-Brody S, Kuja-Halkola R, Sullivan PF, Landén M, Lichtenstein P (2016). Heritability of perinatal depression and genetic overlap with nonperinatal depression. Am J Psychiatry.

[24] Johnstone SJ, Boyce PM, Hickey AR, Morris-Yates AD, Harris MG (2001). Obstetric risk factors for postnatal depression in urban and rural community samples. Aust N Z J Psychiatry.

[25] Tebeka S, Le Strat Y, Dubertret C (2016). Developmental trajectories of pregnant and postpartum depression in an epidemiologic survey. J Affect Disord.

[26] Tebeka S, Le Strat Y, Mandelbrot L, Benachi A, Dommergues M, Kayem G (2021). Early- and late-onset postpartum depression exhibit distinct associated factors: the IGEDEPP prospective cohort study. BJOG An Int J Obstet Gynaecol.

[27] O’Hara MW, Swain AM (1996). Rates and risk of postpartum depression - a meta-analysis. Int Rev Psychiatry.

[28] Beck CT (2001). Predictors of postpartum depression. An update. Nurs Res.

[29] Yim IS, Tanner Stapleton LR, Guardino CM, Hahn-Holbrook J, Dunkel Schetter C (2015). Biological and psychosocial predictors of postpartum depression: systematic review and call for integration. Annu Rev Clin Psychol.

[30] Norhayati MN, Nik Hazlina NH, Asrenee AR, Wan Emilin WMA (2015). Magnitude and risk factors for postpartum symptoms: a literature review. J Affect Disord.

[31] Xiao-hu Z, Zhi-hua Z (2020). Risk factors for postpartum depression: an evidence-based systematic review of systematic reviews and meta-analyses. Asian J Psychiatr.

[32] Hutchens BF, Kearney J (2020). Risk factors for postpartum depression: an umbrella review. J Midwifery Womens Health.

[33] Nisar A, Yin J, Waqas A, Bai X, Wang D, Rahman A (2020). Prevalence of perinatal depression and its determinants in mainland China: a systematic review and meta-analysis. J Affect Disord.

[34] Dadi AF, Akalu TY, Baraki AG, Wolde HF (2020). Epidemiology of postnatal depression and its associated factors in Africa: a systematic review and meta-analysis. PLoS One.

[35] Moher D, Shamseer L, Clarke M, Ghersi D, Liberati A, Petticrew M (2015). Preferred Reporting Items for Systematic Review and Meta-Analysis Protocols (PRISMA-P) 2015 statement. Syst Rev.

[36] Shamseer L, Moher D, Clarke M, Ghersi D, Liberati A, Petticrew M (2015). Preferred Reporting Items for Systematic Review and Meta-Analysis Protocols (PRISMA-P) 2015: Elaboration and explanation. BMJ..

[37] Covidence systematic review software, Veritas Health Innovation, Melbourne, Australia. Available at www.covidence.org.

[38] Kellermeyer L, Harnke B, Knight S (2018). Covidence & Rayyan. J Med Libr Assoc.

[39] Senn SJ (2009). Overstating the evidence - Double counting in meta-analysis and related problems. BMC Med Res Methodol.

[40] Wells G, Shea B, O’Connell D, Peterson J, Welch V, Losos M (2009). The Newcastle-Ottawa Scale (NOS) for Assessing the Quality of Nonrandomised Studies in Meta-Analyses.

[41] Higgins JPT, Li T, Deeks JJ (editors). Chapter 6: Choosing effect measures and computing estimates of effect. In: Higgins JPT, Thomas J, Chandler J, Cumpston M, Li T, Page MJ, Welch VA (editors). Cochrane Handbook for Systematic Reviews of Interventions version 6.3 (updated February 2022). Cochrane. 2022. Available from www.training.cochrane.org/handbook.

[42] Huedo-Medina TB, Sanchez-Meca J, Marin-Martinez F, Botella J (2006). Assessing heterogeneity in meta-analysis: Q statistic or I2 index?. Psychol Methods.

[43] Sterne JAC, Egger M (2001). Funnel plots for detecting bias in meta-analysis: guidelines on choice of axis. J Clin Epidemiol.

[44] Dwan K, Gamble C, Williamson PR, Kirkham JJ (2013). Systematic review of the empirical evidence of study publication bias and outcome reporting bias - an updated review. PLoS One.

[45] Campbell M, McKenzie JE, Sowden A, Katikireddi SV, Brennan SE, Ellis S (2020). Synthesis without meta-analysis (SWiM) in systematic reviews: Reporting guideline. BMJ..

[46] Foroutan F, Guyatt G, Zuk V, Vandvik PO, Alba AC, Mustafa R (2020). GRADE Guidelines 28: Use of GRADE for the assessment of evidence about prognostic factors: rating certainty in identification of groups of patients with different absolute risks. J Clin Epidemiol.

[47] Iorio A, Spencer FA, Falavigna M, Alba C, Lang E, Burnand B (2015). Use of GRADE for assessment of evidence about prognosis: rating confidence in estimates of event rates in broad categories of patients. BMJ..

